# Assessing strain in arrhythmogenic right ventricular cardiomyopathy using cine DENSE MRI

**DOI:** 10.1186/1532-429X-11-S1-P167

**Published:** 2009-01-28

**Authors:** Joash N Ongori, Neil Hendricks, Bruce S Spottiswoode, Ernesta M Meintjes, Frederick H Epstein, Bongani M Mayosi

**Affiliations:** 1 MRC/UCT Medical Imaging Research Unit, University of Cape Town, Cape Town, South Africa; 2 Cardiac Clinic and Department of Medicine, University of Cape Town, Cape Town, South Africa; 3 Biomedical Engineering and Radiology, University of Virginia, Charlottesville, VA USA

**Keywords:** Right Ventricle, Arrhythmogenic Right Ventricular Cardiomyopathy, Tangential Strain, Right Ventricle Free Wall, Anterior Septum

## Introduction

The kinematics of the right ventricle (RV) are not well understood due to its thin wall and asymmetric geometry [1]. Cine displacement encoding with stimulated echoes (cine DENSE), which measures intramyocardial displacement and strain [2], has been adapted for RV analysis with encouraging preliminary results in normal subjects [3]. This preliminary study evaluates cine DENSE MRI for detecting decreased myocardial strain in arrhythmogenic right ventricular cardiomyopathy (ARVC). ARVC is a unique heart muscle disease affecting predominantly the RV. The pathological hallmark of fibro-fatty replacement of the myocardium may result in localised aneurysms and wall motion abnormalities, detectable by cardiac magnetic resonance imaging [4].

## Methods

In accordance with protocols approved by the UCT Research Ethics Committee, 3 confirmed, 6 suspected ARVC cases and 4 normal volunteers were scanned using cine DENSE. ARVC diagnosis was based on standard clinical criteria [4]. Cine DENSE images were acquired in basal, mid and apical short axis slices on a Siemens 1.5 T Symphony scanner using segmented echo planar imaging for data acquisition [2]. Displacement encoding was applied in two orthogonal in-plane directions over two breath holds per slice. Imaging parameters include: FOV = 400 mm, slice thickness = 7.0 mm, TR = 24 ms, FOV phase = 62.5%, ETL = 9, segments = 18, cardiac phases = 10–16, and displacement encoding frequency = 0.1 cycles/mm. Epicardial and endocardial contours were manually drawn for both the LV and RV on all cardiac phases. Phase unwrapping and tissue tracking were performed [5], and motion trajectories were estimated using temporal fitting with 5^th^ order Polynomial functions. Lagrangian strain taken tangential to the midwall (Ett) was computed from both the left ventricle (LV) and RV motion trajectories [3].

## Results

**Figure 1:**
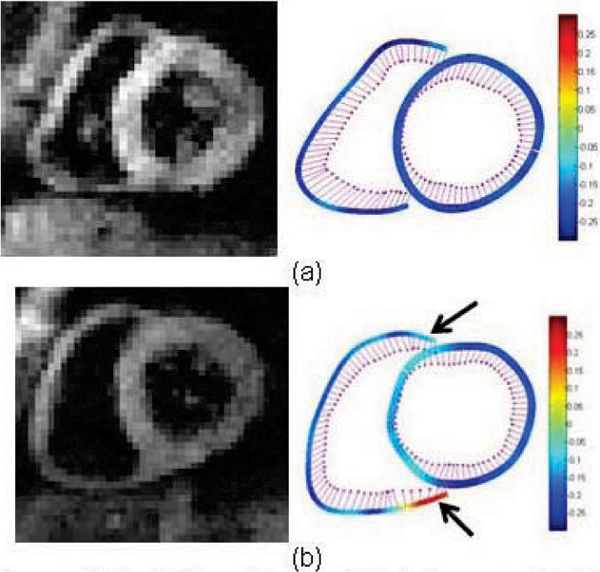
**Cine DENSE magnitude** ***(left)*** **and tangential strain maps** ***(right)*** **for a normal volunteer (a), and one confirmed ARVC case (b)**.

Example basal slice cine DENSE magnitude images and corresponding end systolic tangential strain maps are shown for a normal volunteer (Figure 1a) and a confirmed ARVC case (Figure 1b). The underlying colour represents Ett and the purple vectors represent displacement. The arrows in Figure 1b indicate regions of abnormal strain and motion, which occur largely in the region of the RV insertion points and the LV inferior and anterior septum. Abnormal RV strain patterns were evident in 2 out of the 3 confirmed, and 3 out of the 6 suspected cases. The other confirmed case had a dilated RV but the strain pattern appeared normal. Table 1 presents regional peak tangential strains for the basal slice averaged for each group. A confidence measure for Ett was calculated based on the signal-to-noise ratio (SNR) of the magnitude images. Only Ett strain values with confidence greater than 0.5 (normalized to max confidence in entire heart) were used to compute regional peak Ett strain. More than half of the data points in the diaphragmatic RV had to be discarded because of low SNR. Both confirmed and suspected ARVC cases have low peak strain values in the inferior and anterior septum and the RV free wall. Peak Ett is significantly less in ARVC and suspected ARVC cases compared to normals in the anterior septum (p < 0.005 and p < 0.05, respectively, student t-test).

**Table 1:** Basal slice mean peak tangential strain values and standard deviation for three confirmed ARVC cases, six suspected cases, and four normal volunteers for the LV and RV segments.

Peak tangential strains						
	Confirmed ARVC cases		Suspected ARVC cases		Normals	
Regions	Mean	Std Dev	Mean	Std Dev	Mean	Std Dev
Anterior LV wall	-0.11	0.04	-0.11	0.05	-0.16	0.03
Lateral LV wall	-0.15	0.04	-0.14	0.05	-0.17	0.03
Posterior Lvwall	-0.16	0.03	-0.15	0.06	-0.19	0.02
Inferior LV	-0.15	0.06	-0.15	0.05	-0.18	0.04
Inferior septum	-0.11	0.03	-0.11	0.03	-0.16	0.03
Anterior septum	-0.08	0.02	-0.11	0.04	-0.15	0.02
RV free wall	-0.15	0.03	-0.13	0.04	-0.18	0.02
Diaphragmatic RV	-0.13	0.01	-0.10	0.02	-0.13	0.04

## Conclusion and discussion

Preliminary results indicate that cine DENSE CMR is useful in detecting abnormal myocardial strain in ARVC. Due to improved SNR and motion properties, cine DENSE using spiral data acquisition [6] may improve the ability to measure strain in the diaphragmatic RV segment.
